# Predictors of neurocognition outcomes in children and young people with primary brain tumor presenting to tertiary care hospitals of Karachi, Pakistan: a prospective cohort study

**DOI:** 10.1007/s00381-024-06306-x

**Published:** 2024-02-16

**Authors:** Nida Zahid, S. Ather Enam, Thomas Mårtensson, Iqbal Azam, Naureen Mushtaq, Mariya Moochhala, Farrukh Javed, Faiza Kausar, Aneesa Hasan, Lal Rehman, M. Nouman Mughal, Sadaf Altaf, Salman Kirmani, Nick Brown

**Affiliations:** 1 Department of Surgery, Aga Khan University, Karachi, Pakistan; 2 Global Health and Migration Unit, Department of Women’s and Children’s Health, Uppsala University, Uppsala, Sweden; 3 Department of Community Health Sciences, Aga Khan University, Karachi, Pakistan; 4 Department of Pediatric Oncology, Aga Khan University, Karachi, Pakistan; 5 Department of Psychiatry, Aga Khan University, Karachi, Pakistan; 6 Department of Neurosurgery, Jinnah Postgraduate Medical Centre, Karachi, Pakistan; 7 Division of Women & Child Health, Aga Khan University, Karachi, Pakistan; 8 Department of Pediatrics, Aga Khan University, Karachi, Pakistan

**Keywords:** Neurocognition outcomes, Children and young people, Primary brain tumor, Cohort study, Pakistan

## Abstract

**Introduction:**

Primary brain tumors are a common cause of morbidity and mortality in children and young people (CYP) globally. Impaired neurocognitive function is a potential severe consequence in primary brain tumor (PBT) survivors. There are no in-depth studies from low- and middle-income countries (LMICs) to inform management and follow-up. The research questions of this study were as follows: Are the sociodemographic factors (lower age of CYP, female gender, low socioeconomic status, low parental education), disease-related factors (high grade of tumor, presence of seizures, presence of hydrocephalous), and treatment-related factors (adjuvant therapy, no surgical intervention, post-treatment seizures, placement of shunts) associated with decline in neurcognition outcomes 12 months post-treatment in CYP with PBTs?

**Methods:**

A prospective cohort study was conducted from November 2020 to July 2023 at the Aga Khan University Hospital and Jinnah Postgraduate Medical Centre, tertiary care hospitals in Karachi, Pakistan. All CYP aged 5 to 21 years with a newly diagnosed PBTs were eligible. The neurocognition assessment was undertaken by a psychologist at two points, i.e., pre-treatment and at 12 months post-treatment using validated tools. The verbal intelligence was assessed by Slosson Intelligence tool, revised 3rd edition (SIT-R3), perceptual reasoning by Raven’s Progressive Matrices (RPM), and the Processing Speed Index by Wechsler Intelligence Scale (WISC V) and Wechsler Adult Intelligence Scale (WAIS-IV). The data were analyzed by STATA version 12 software. Generalized estimating equation (GEE) was used to determine the factors associated with the mean change in 12 months post-treatment verbal and non-verbal neurocognition scores. Unadjusted and adjusted beta coefficients with their 95% confidence intervals were reported.

**Results:**

A total of 48 CYPs with PBTs were enrolled, 23 (48%) of them were lost to follow-up and 10 (21%) died. The remaining 25 (52%) were reassessed 12 months after treatment. On multivariable analysis, a significant decline in verbal intelligence scores at 12 months was predicted by post-treatment seizures beta =  − 20.8 (95% CI, − 38.2, − 3.4), mothers having no formal educational status and lower household monthly income. Similarly, a significant decline in perceptual reasoning scores was also predicted by post-treatment seizures beta =  − 10.7 (95% CI, − 20.6, − 0.8), mothers having no formal education and having lower household monthly income. Worsening of processing speed scores at 12 months post-treatment were predicted by tumor histology, post-treatment seizures beta =  − 33.9 (95% CI, − 47.7, − 20.0), lower educational status of the mother, and having lower household monthly. However, an improvement was seen in processing speed scores after surgical tumor resection.

**Conclusion:**

In this novel study, the post-treatment mean change in verbal and non-verbal neurocognition scores was associated with sociodemographic, tumor, and treatment factors. These findings may have potential implications for targeted early psychological screening of higher risk CYP with PBTs. Identification of these predictors may serve as a foundation for developing more cost-effective treatment thereby alleviating the burden of neurocognitive morbidity. However to establish generalizability, future research should prioritize larger-scale, multicountry studies. (Trial registration: ClinicalTrials.gov Identifier: NCT05709522)

**Supplementary Information:**

The online version contains supplementary material available at 10.1007/s00381-024-06306-x.

## Introduction

Globally, primary brain tumors (PBTs) are the second most common type of cancer in children and young people (CYP) aged 0–24 years [1]. The World Health Organization (WHO) estimated in 2020 that approximately 10% of all primary brain tumors (PBTs) occur in children and adolescents, ages 0–19 years, and 7% of all PBTs occur in the young adult population aged 20–24 years [2]. Almost 90% of these are from low- and middle-income countries (LMICs) [3]. Anatomically, approximately 60% of the PBTs are located in the posterior fossa the part of the brain under the tentorium and which includes the cerebellum, brainstem, and fourth ventricle, in children 0–15 years [4].

The survival rate of PBTs is increasing due to improvements in detection and treatment. It was reported by the WHO in 2020 that the 5-year relative mortality for all PBTs (0–19 years of age) was 14% [2]. The corresponding figure for young adults (20–24 years of age) was 10% [2]. In 2020, a noteworthy decrease of 14% in mortality rate was observed when compared to 2010 [5]. However, improved survival has been associated with adverse late effects [6–9].

The term “adverse late effects” encompasses the cumulative negative consequences or unfavorable outcomes that develop over time following the initial treatment. These effects can manifest as a decline in physical, cognitive, emotional, and functional well-being, leading to a reduction in an individual’s overall quality of life (QoL). Often, these negative consequences become apparent months or even years after the initial diagnosis and treatment of tumor [10].

Work in this area suggest that late effects occur in 6 to 21% of childhood cancer survivors aged 0 to 18 years at diagnosis [11, 12]. One of the most devastating consequences is the impairment of neurocognitive functioning from critical thinking, problem-solving, executive control, memory retention, and focused attention. These range from mild to severe, often persisting or even worsening with time (9). It is estimated that 20 to 50% of children aged (6–16 years) with central nervous system (CNS) tumors demonstrate impairment in at least one neurocognitive domain [13]. However, by implementing specialized educational services, precise therapeutic interventions, and customized classroom accommodations, it is possible to significantly alleviate the impact of neurocognitive impairment [10].

A narrative review by Stavinoha et al. showed evidence from a range of studies from high-income countries of the neurocognitive and psychological outcomes in PBT survivors. The studies making up this review, however, were largely cross-sectional or, where longitudinal, without a clearly defined baseline [14]. A study conducted in Canada assessed cognitive outcomes during long-term follow-up among pediatric brain tumors survivors, with repeat testing 1–3 years after initial testing, and estimated a mean decline of a range of cognitive outcomes between 1 and 1.7 standard deviation scores from the baseline pre-treatment values [15]. A meta-analysis of 39 empiric studies reported that these CYP exhibited significant and pervasive impairments in multiple neurocognitive domains and that survivors’ scores on measures of global cognitive ability, verbal and nonverbal, fell nearly 1 standard deviation below normative means [16]. Poon et al. conducted a systematic review including 59 studies assessing the late outcome of childhood cancer in Asian countries [17]. The majority of the relevant studies included survivors of CNS tumors and pediatric acute lymphoblastic leukemia (ALL) [17]. However, only one assessed the neurocognition outcomes in CNS tumor survivors reporting neurocognitive impairment in 10% of the survivors [18].

Importantly, to best of the investigator’s knowledge, there are no in-depth studies evaluating neurocognitive outcome including CYPs with primary brain tumor from low- and middle-income countries (LMICs). Only one study from a tertiary care hospital in Karachi, Pakistan, assessed the neurological, endocrine, hypothalamic complications and survival outcomes in children with craniopharyngioma. However, this study did not assess the neurocognitive outcomes [19]. Due to difference in healthcare infrastructure, socioeconomic factors, cultural differences, and access to advanced diagnostics and treatments in this region, data from high-income countries cannot be extrapolated.

This study included CYP, 5–21 years of age, referred to two tertiary care hospitals in Karachi, Pakistan, and assessed neurocognitive function pre-treatment and reassessed at 12 months post-treatment. The aim of the study was to identify sociodemographic, birth, and disease- and treatment-related factors associated with neurocognitive impairment and deterioration after treatment.

Research questions:

1. Does sociodemographic factors of CYP such as lower age, female gender, and low socioeconomic status cause a decline in the mean neurocognition scores 12 months post-treatment?
2. Does parental sociodemographic factors such as low educational status of parents and low household monthly income cause a decline in the mean neurocognition scores 12 months post-treatment?
3. Are the CYP tumor factors such as high grade of tumor, presence of seizures, and presence of hydrocephalous associated with decline in neurocognition outcomes 12 months post-treatment?
4. Are the CYP treatment factors such as adjuvant therapy, no surgical intervention, post-treatment seizures, and placement of shunts associated with decline in neurcognition outcomes 12 months post-treatment?

## Methods

### Study design and setting

A prospective cohort study was conducted from November 2020 to July 2023 at the Aga Khan University Hospital (AKUH), a Joint Commission International Accreditation (JCIA‐accredited) private sector tertiary care hospital, in Karachi, Pakistan, and Jinnah Postgraduate Medical Centre (JPMC) a public sector tertiary care hospital, in Karachi, Pakistan.

### Study population and eligibility criteria

All CYP, aged 5–21 years, residing in Pakistan with PBTs, presenting at any stage, without previous treatment were eligible. Participants with any known history of psychiatric or neurological illness such as attention deficit hyperactivity disorder (ADHD), autism, and schizophrenia, confirmed by the medical records, or with physical morbidities, were excluded. Children without formal education were also excluded based on the assumption that they might have limited mathematical and language skills, limiting objective evaluation of their cognitive abilities. CYP with complete pre- and post-treatment data were included in the final regression analysis.

### Sampling technique

A non-probability purposive sampling technique was employed for selecting the participants. A trained research assistant approached all the CYP with newly diagnosed PBTs presenting to surgical/oncology clinics at AKUH and JPMC during their scheduled appointments. Potential participants were screened for eligibility. If eligible, potential participants and their parents were briefed regarding the scope and nature of the study, as well as the extent of their participation. They were included if patients’ written assent/informed consent and parents’ written consent were provided.

### Sample size

The sample size was calculated using standard estimate where *n* = 8 (CV^2^)/(PC^2^) [1 + (1 − PC)^2^] where PC is the proportionate change in means (PC = (µ0 − µ1)/µ0) and CV is the coefficient of variation (CV = σ0/µ0 = σ1/µ1) [20]. To allow for an inflation rate of 65% (based on previous work) [3], a minimum sample size of 48 CYP with PBTs was estimated to achieve 80% power and to detect at least a 15% change in mean neurocognition scores and 19% or less change in coefficient of variation as reported by Shortman et al., at two sided 5% level of significance [21].

### Data collection procedure

Prior to participant’s recruitment, the questionnaire and the neurocognitive tools were pre-tested on 5% of the participants to assess the feasibility. A trained research assistant administered the final questionnaire in Urdu (the national language and official language of Pakistan). Data were gathered on the following variables:

#### Independent variables

CYP's demographic information including (age, educational status, gender, etc.); parent’s demographic information including (age, educational status, household monthly income); tumor and treatment information that included tumor histopathology and tumor location (supratentorial, infratentorial, suprasellar, and sellar); history of pre-treatment seizures; the presence or absence of hydrocephalus at diagnosis confirmed radiologically by magnetic resonance imaging (MRI); type of treatment (surgical resection, chemotherapy, radiotherapy, and biopsy); and type of surgery , i.e., total resection (100% removal of tumor), maximum safe resection (> 90% removal of tumor while prioritizing safety), and subtotal resection (< 90% removal of tumor), these definitions are based on institutionally established cutoffs, determined through post-MRI assessments of residual tumor. Management by external ventricular drain (EVD) and ventriculoperitoneal shunt (VPS) for those with hydrocephalous at diagnosis.

#### Outcome variable

##### Neurocognition

The neurocognition assessment was undertaken by the same psychologist at the pre-treatment and 12 months post-treatment stages. The assessments were undertaken at both the study sites in English or Urdu depending on the understanding and preference of the patient and/or parents.

The verbal intelligence was assessed by Slosson Intelligence tool, revised 3rd edition (SIT-R3). The SIT-R total standard scores were presented as standard scores with a mean (M) of 100 and a standard deviation (SD) of 16. A higher score on the Slosson indicated a more favorable outcome [22]. Although the tool has been translated and adapted for use in Pakistan [23], validation in Urdu is lacking. To address this, a content validity index (CVI) was computed by a panel of experts. These experts assessed the tool for relevance and clarity in relation to the Pakistani cultural context, using a Likert scale of 1 to 4. The CVI scores, which are the average of the content validity ratio (CVR) for each item, were used to determine agreement. The relevance CVI was estimated at 0.90, and the clarity CVI was 0.89. These results reflect a high level of agreement among experts regarding the clarity and relevance of the tool’s content.

Perceptual reasoning was assessed by Raven’s Progressive Matrices (RPM) [24]; a higher score on the RPM indicated a more favorable outcome. It has been previously validated in Pakistan [25].

The Processing Speed Index (PSI) was assessed by Wechsler Intelligence Scale (WISC V) [26, 27] and Wechsler Adult Intelligence Scale (WAIS-IV) [28]. This has been previously validated in Pakistan [29]. A higher score indicated a more favorable outcome.

### Ethical considerations

Ethical/institutional review committee approvals from the respective study sites were obtained, AKUH-ERC (ERC#2020–4859-11855) and JPMC (F2-81/2021-GENL/65706/JPMC). Written informed consent was taken directly from patients aged 18 years and older. For those under 18 years of age, both informed assent and parental consent was obtained in English or Urdu as per the understanding of the participants. To ensure confidentiality, interviews were conducted in a separate room. The psychologist provided treatment plan to those identified with a 12 months post-treatment neurocognitive decline at both the study sites.

### Statistical analysis

Data were analyzed using STATA version 12 (Stata Texas^®^). Results were presented as mean and standard deviation (SD)/median and interquartile range (IQR) for quantitative variables. The difference in mean pre-treatment and 12 months post-treatment neurocognitive scores was assessed by paired *t*-test. Categorical variables were reported as frequency and percentages and were assessed by chi-square or Fisher’s exact test. To determine the association of various independent factors such as child factors (demographic factors), parental factors (educational status, socioeconomic status), and tumor and treatment-related factors with the change in mean neurocognitive scores, generalized estimating equation was applied and the covariates were adjusted. Beta coefficient (unadjusted and adjusted) with 95% confidence interval (CI) was reported. Multicollinearity and plausible interactions were assessed. A *p*-value of less than 0.2 at univariate analysis and less than 0.05 at multivariable analysis were considered statistically significant.

## Results

**Fig. 1:**
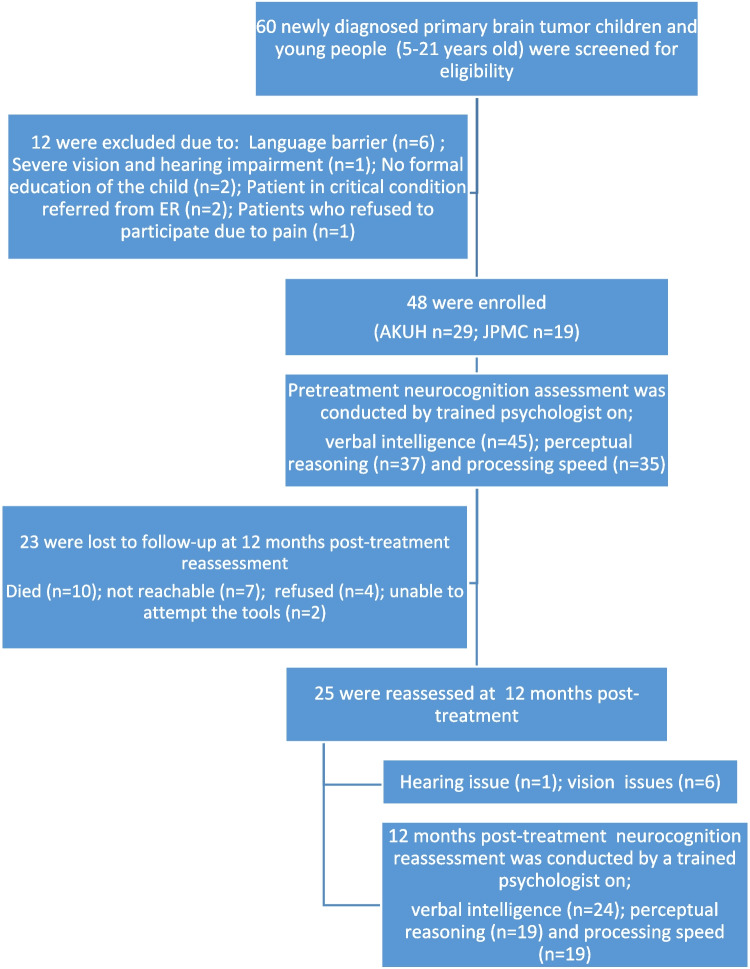
Flow of the study

A total of 60 CYP with PBTs were screened for eligibility. We excluded 12 participants. The remaining 48 were enrolled. Among those, 23 (48%) were lost to follow-up, of whom 10 died. At 12 month post-treatment, 25 participants were reviewed. Within this group, one had hearing issues, and six had vision issues. Consequently, the follow-up data on verbal intelligence was obtained from 24 participants, data on perceptual reasoning from 19 participants, and data on processing speed from 19 participants (Fig. 1).

### Sociodemographic and birth-related factors

The mean age of the recruited CYP was 12.8 (4.6) years. Sixty percent were males. The median household monthly income was 106 USD (Table 1).

**Table 1:** Sociodemographic and birth-related factors of children and young people 5–21 years (CYP) with primary brain tumor (*n* = 48)

**Characteristics**	***n*** **(%)**
**Demographics**	
**Patient’s age (in years)**	
5–9	15 (31)
10–14	14 (29)
15–17	11 (23)
18–21	8 (17)
**Mean age of the patients in years (SD)**	12.8 (4.6)
**Gender**	
Male	29 (60)
Female	19 (40)
**Province of residence**	
Sindh	35 (73)
Punjab	8 (17)
Khyber Pakhtunkhwa	4 (8)
Gilgit-Baltistan	1 (2)
**Mother tongue**	
Sindhi	7 (15)
Urdu	22 (46)
Pushto	5 (10)
Punjabi	8 (17)
Others (Saraiki and Shina)	6 (12)
**Educational status of the CYP**	
Primary	24 (50)
Secondary	15 (31)
Higher secondary or above	9 (19)
**Median years of CYP’s formal education (IQR)**	5.5 (1.3–9.8)
**Household Family Members**	
** ≤ 6**	21 (44)
** > 6**	27 (56)
**Median number of household family members (IQR)**	7 (5–9)
**Number of siblings**	
≤ 3	31 (65)
> 3	17 (35)
**Median number of siblings (IQR)**	3 (2–4)
**Birth factors**	
Gestational age*	
Preterm	7 (15)
Term	41 (85)
**Mean gestational age in weeks (SD)**	37.2 (2.1)
**Birth order**	
First	15 (31)
Middle	25 (52)
Last	8 (17)
**Median birth order of the patients (IQR)**	2 (1–3)
**Parental sociodemographics**	
**Age of mothers (in years)**	
25–34	16 (33)
≥ 35	32 (67)
**Mean age in years (SD)**	37.9 (6.6)
**Age of fathers (in years)**	
25–34	5 (10)
≥ 35	43 (90)
**Mean age in years (SD)**	42.7 (7.9)
**Marital status**	
Married	42 (88)
Others (widower, widow, divorce)	6 (12)
**Educational status of mothers**	
No formal education	12 (24)
Primary	9 (19)
Secondary	8 (17)
Higher secondary and above	19 (40)
**Median years of mother’s education (IQR)**	9 (0.3–12)
**Education status of fathers**	
No formal education	10 (21)
Primary	3 (6)
Secondary	9 (19)
Higher secondary and above	26 (54)
**Median years of father’s education (IQR)**	12 (5–14)
**Working status of the parents**	
Only father working	38 (79)
Only mother working	2 (4)
Both father and mother working	3 (6)
Both father and mother not working	5 (11)
**Household monthly income (in USD)**	
≤ 53	8 (17)
53–159	23 (48)
159–320	7 (15)
> 320	10 (20)
**Median monthly income in USD (IQR)**	106 (70.7–282.7)

Current conversion rate of US dollars (USD) to Pakistan Rupee (PKR) is 283

^*^Gestational age; preterm defined as delivery before 37 weeks of gestation; term defined as delivery between 37 + 0 and 41 + 6 weeks

### Tumor and treatment-related factors

There was no significant difference in CYP's mean age at diagnosis 12.5 (4.5) years and at presentation to the hospital for treatment 12.8 (4.6) years. Twenty two out of forty eight (46%) required a ventriculoperitoneal shunt (VPS) or external ventricular drain (EVD). Thirty eight out of 48 (79%) underwent surgery (Table 2).

**Table 2:** Tumor and treatment factors in children and young people (5–21 years) with primary brain tumor (*n* = 48)

**Characteristics**	***n*** **(%)**
**Site of treatment**	
Private tertiary care hospital	29 (60)
Public tertiary care hospital	19 (40)
**Location of primary brain tumor**	
**Supratentorial**	12 (25)
Cerebrum	10
Intraventricular	2
**Infratentorial**	17(35)
Cerebellum	8
Brainstem	6
Fourth Ventricle	3
**Suprasellar**	12(25)
Hypothalamus	4
Thalamus	2
Third ventricle	2
Infundibulum	1
Optic chiasma	2
Not known	1
**Sellar**	5(10)
Pituitary	5
**Multiple**	2(5)
**Tumor histopathology**	
Glioblastoma	4 (8)
Medulloblastoma	7 (15)
Ependymoma	3 (6)
Diffuse astrocytoma	1 (2)
Craniopharyngioma	5 (10)
Pilocytic astrocytoma	12 (25)
Pituitary adenoma	5 (10)
Others^a^	3 (6)
Not known	8 (18)
**Grade of tumor**	
Grade I	24 (50)
Grade II	3 (6)
Grade III	2 (4)
Grade IV	11 (23)
Not known	8 (17)
**Tumor size (in cm**^**3**^)	
≤ 9.1	8 (17)
9.1–35	8 (17)
35–90	8 (17)
> 90	7 (15)
Not available	17 (34)
**Median tumor size (IQR)**	35,088 (9135–90,000)
**History of seizure**	
Yes	10 (21)
No	38 (79)
**Hydrocephalus**	
Present	23 (48)
Absent	25 (52)
**Family history of brain tumor**	
Yes	5 (10)
No	43 (90)
**Family history of any other cancer**	
Yes	11 (23)
No	37 (77)
**Post-treatment seizures(n=25)**	
Yes	4 (16)
No	21 (84)
**Type of treatment**	
Surgery only	26 (54)
Radiotherapy only	1 (2)
Combination	12 (25)
Surgery and chemotherapy	4
Surgery and radiotherapy	3
Surgery, radiotherapy, and chemotherapy	5
No intervention	9 (19)
**Type of surgery**^**b**^	
Biopsy	1 (2)
Total resection	10 (21)
Subtotal resection	2 (4)
Maximum safe resection	25 (52)
No surgical intervention	10 (21)
**Presence of VPS/EVD**	
Yes	22 (46)
Only EVD	4
Only VPS	10
EVD and VPS	8
No	26 (54)
**Tumor recurrence (at 12 months post-treatment follow-up)**	
Yes	1 (2)
No	25 (52)
No information	22 (46)
**Alive (at 12 months post-treatment follow-up)**	
Yes	31 (65)
No	10 (21)
No information	7 (14)
**Median months of overall survival (IQR)**	4.5 (1.3, 11.8)

^*^

*EVD* external ventricular drain, *VPS* ventriculoperitoneal shunt, *IQR* interquartile range

^a^Histopathology for others include anaplastic astrocytoma, optic chiasma glioma, and choroid plexus papilloma

^b^Type of surgery: total resection (100% of tumor removal), maximum safe resection (> 90% of tumor removal), subtotal resection (< 90% of tumor removal)

Post-treatment seizures were reported in 4 out of 25 (16%) CYP. A higher percentage with low-grade tumors (33%) versus high-grade tumors (7%) underwent total resection. After treatment, 14 out 25 (56%) discontinued their education, and 11 out 14 (79%) who discontinued were those who belonged to households with monthly income of less than USD 159. Among the 10/48 (21%) patients who expired, 5 out of 10 (50%) had medulloblastoma, 1 out 10 (10%) had pituitary adenoma, and 1 out of 10 (10%) had craniopharyngioma (Table 2).

### Pre-treatment neurocognition scores

The pre-treatment mean verbal neurocognitive score (81 ± 19.5) was 1 standard deviation (SD) below the normative mean, while the pre-treatment mean perceptual reasoning score (94 ± 15.3) was 0.41 SD below the normative mean and the mean processing speed (63 ± 14.4) was 2 SD below the normative mean. There was statistically significant positive correlation between verbal intelligence score and perceptual reasoning score (*r* = 0.5; *p* = ≤ 0.002). Similarly, a significant positive correlation was also observed between verbal intelligence score and processing speed score (*r* = 0.5; *p* = 0.001). Furthermore, a significant positive correlation was observed between perceptual reasoning score and process speed score (*r* = 0.4; *p*-value = 0.047). The verbal intelligence score of 70% of the participants was below average, while perceptual reasoning score of 44% of the participants was below average (Supplementary 1).

### Post-treatment neurocognition scores

The post-treatment mean verbal intelligence score (84 ± 22.9) was one standard deviation below the normative mean. The post-treatment mean perceptual reasoning score (92 ± 14.7) was 0.61 standard deviation below the normative mean, and the post-treatment mean processing speed score (70 ± 15.5) was 2 SD below the normative mean. No statistically significant difference was observed in the mean change in neurocognition scores at 12 months post-treatment reassessment among the participants.

### Univariable analysis

On univariate analysis, those with post-treatment seizures had a statistically significant decline in verbal intelligence, perceptual reasoning, and processing speed scores (*p*-values < 0.2). Furthermore, a statistically significant increase in verbal intelligence scores and processing speed scores was observed in those who underwent surgical resection (*p*-value = 0.1 and *p*-value = 0.01), respectively. Individuals whose parents possessed higher educational qualifications and those who belonged to households with higher monthly income had a significant improvement in verbal intelligence, perceptual reasoning, and processing speed scores (*p*-values < 0.001). A significant decline was observed in verbal intelligence and processing speed scores in patients with tumor histopathology of ependymoma, craniopharyngioma, glioblastoma, and medulloblastoma (*p*-values ≤ 0.2). Finally, those with hydrocephalous had a significant decline in processing speed scores (*p*-value = 0.1) (Table 3).

**Table 3:** Factors associated with mean change in neurocognition scores among children and young people (5–21 years) with primary brain tumor

**Characteristics**	**Verbal intelligence (*n*** = 24)	**Perceptual reasoning (*n* = 19)**	**Processing speed (***n*** = 19)**
	** Unadjusted β coefficient (95% CI)**		
**Tumor histopathology**			
Pilocytic astrocytoma	(Reference)		(Reference)
Ependymoma	− 21.0 (− 45.1, 3.2)		− 22.2 (− 41.4, − 3.0)
Craniopharyngioma	− 11.1 (− 30.7, 8.6)		− 13.7 (− 27.1, − 0.3)
Glioblastoma	− 2.4 (− 23.7, 19.0)	-	− 14.6 (− 29.6, 0.5)
Medulloblastoma	− 17.8 (− 35.6, 0.03)		− 18.9 (− 31.7, − 6.1)
Pituitary adenoma	− 21.8 (− 41.6, − 1.9)		− 2.8 (− 16.5, 11.0)
Others	− 8.7 (− 32.4, 15.0)		− 5.9 (− 19.6, 7.9)
**Grade of tumor**			
Low	-	-	(Reference)
High			− 7.5 (− 17.9, 3.0)
**Post-treatment seizures**			
Yes	− 18.1 (− 39.4, 3.3)	− 6.6 (− 20.3, 7.1)	− 13.9 (− 27.3, − 0.5)
No	(Reference)	(Reference)	(Reference)
**Surgery intervention**^**a**^			
No intervention	(Reference)		(Reference)
Total resection	12.8 (− 4.6, 30.1)	-	12.7 (0.8, 24.5)
Subtotal resection	− 18.8 (− 48.4, 10.8)		− 13.6 (− 37.5, 10.4)
Maximum safe resection	3 (− 11.9, 17.8)		0.3 (− 10.4, 11.0)
**Hydrocephalous**			
Yes	-	-	− 5.78 (− 14.4, 2.8)
No			(Reference)
**Discontinued school after treatment**			
No	Reference	(Reference)	Reference
Yes	− 17.8 (− 32.6, − 3.0)	− 6.5 (− 15.8, 2.8)	− 15.3 (− 24.1, − 6.6)
**Number of siblings**			
≤ 3	(Reference)	-	-
> 3	− 20.1 (− 31.6 − 8.5)		
**Household family members**			
**≤ 6**	(Reference)	-	-
**> 6**	− 23.1 (− 33.6, − 12.7)		
**Educational status of fathers**			
No formal education	− 22.4 (− 36.7, − 8.1)	-	-
Primary	− 24.5 (− 47.2, − 1.8)		
Secondary	− 10.8 (− 25.1, 3.5)		
Higher secondary and above	(Reference)		
**Educational status of mothers**			
No formal education	− 28.5 (− 41.6, − 15.5)	− 11.7 (− 21.6, − 1.8)	− 4.6 (− 15.6, 6.4)
Primary	− 27.7 (− 41.7, − 13.8)	− 14.5 (− 26.1, − 3.0)	− 10.1 (− 22.1, 2.0)
Secondary	− 14.8 (− 29.2, − 0.3)	− 1.5 (− 11.1, 8.1)	− 2.0 (− 13.7, 9.7)
Higher secondary and above	(Reference)	(Reference)	(Reference)
**Age of fathers (years)**			
25–34	(Reference)	-	(Reference)
≥ 35	− 16.9 (− 34.9, 1.2)		− 14.4 (− 30.2, 1.5)
**Household monthly income (in USD)**			
≤ 53	− 34.4 (− 51.4, − 17.5)	− 15.5 (− 28.0, − 2.9)	− 24.1 (− 35.8, − 12.5)
53–159	− 22.7 (− 35.8, − 9.5)	− 9.4 (− 18.3, − 0.5)	− 9.3 (− 18.1, − 0.4)
159–320	− 24.0 (− 41.0, − 7.0)	− 14.3 (− 25.2, − 3.4)	− 8.7 (− 19.7, 2.3)
> 320	(Reference)	(Reference)	(Reference)
**Household monthly income (in USD)**	0.01 (0.01, 0.02)	-	%1.%2 (0.002, 0.01)

^a^

^a^Total resection (100% of tumor removal), maximum safe resection (> 90% of tumor removal), subtotal resection (< 90% of tumor removal)

*CI* confidence interval

### Multivariable analysis

On multivariable analysis, a statistically significant decline in verbal intelligence at 12 months was predicted by post-treatment seizures beta =  − 20.8 (95% CI, − 38.2, − 3.4), mothers having no formal education beta =  − 18.2 (95% CI, − 33.7, − 2.8) and having lower household monthly income of < USD 53 beta =  − 29.7 (95% CI, − 47.9, − 11.6) and USD 159–320 beta =  − 21.1 (95% CI, − 39.7, − 2.5) as compared to those with income of > USD 320 (Table 4, Supplementary 2).

**Table 4:** Factors associated with mean change in neurocognition scores among children and young people (5–21 years) with primary brain tumor

**Characteristics**	**Verbal intelligence (*n* = 24)**	**Perceptual reasoning (*n* = 19)**	**Processing speed (*n* = 19)**
	**Adjusted β coefficient (95% CI)**		
**Tumor histopathology**			
Pilocytic astrocytoma			(Reference)
Ependymoma	-	-	− 34.3 (− 57.7, − 11.0)
Craniopharyngioma			− 23.3 (− 39.4, − 7.2)
Glioblastoma			− 35.6 (− 47.5, − 23.6)
Medulloblastoma			− 15.2 (− 31.5, 1.0)
Pituitary adenoma			− 6.2 (− 13.7, 1.2)
Others			− 7.3 (− 17.1, 2.5)
**Post-treatment seizures**			
Yes	− 20.8 (− 38.2, − 3.4)	− 10.7 (− 20.6, − 0.8)	− 33.9 (− 47.7, − 20.0)
No	(Reference)	(Reference)	(Reference)
**Surgery intervention**			
No intervention			(Reference)
Total resection	-	-	16.6 (− 1.3, 34.5)
Subtotal resection			63.3 (27.8, 98.9)
Maximum safe resection			28.9 (10.4, 47.5)
**Educational status of mothers**			
No formal education	− 18.2 (− 33.7, − 2.8)	− 9.8 (− 18.6, − 1.0)	− 7.0 (− 14.8, 0.7)
Primary	− 15.7 (− 36.7, 5.2)	− 10.4 (− 24.8, 4.0)	− 14.6 (− 25.8, − 3.4)
Secondary	− 6.7 (− 24.0, 10.7)	4.7 (− 4.5, 14.0)	− 11.2 (− 19.7, − 2.8)
Higher secondary and above	(Reference)	(Reference)	(Reference)
**Household monthly income**			
≤ 53	− 29.7 (− 47.9, − 11.6)	− 22.0 (− 33.5, − 10.5)	− 30.5 (− 42.3, − 18.7)
53–159	− 12.7 (− 28.6, 3.2)	− 11.9 (− 20.4, − 3.4)	− 5.8 (− 11.5, − 0.1)
159–320	− 21.1 (− 39.7, − 2.5)	− 11.0 (− 20.6, − 1.3)	− 10.5 (− 18.8, − 2.1)
> 320	(Reference)	(Reference)	(Reference)

*CI* confidence interval

Similarly, a statistically significant decline in perceptual reasoning scores was predicted by post-treatment seizures beta =  − 10.7 (95% CI, − 20.6, − 0.8), mothers having no formal education beta =  − 9.8 (− 18.6, − 1.0) and having lower household monthly income of < USD 53 beta =  − 22 (95% CI, 33.5, − 10.5), USD 53–159 beta =  − 11.9 (95% CI, − 20.4, − 3.4), and USD 159–320 beta =  − 11 (95% CI, − 20.6, − 1.3) as compared to those with income of > USD 320 (Table 4, Supplementary 2).

Furthermore, the processing speed scores were significantly decreased at 12 months among participants with the following tumor histopathology: ependymoma, glioblastoma, and craniopharyngioma when compared to pilocytic astrocytoma. Moreover, a statistically significant decline in processing speed scores were  also predicted by post-treatment seizures beta =  − 33.9 (95% CI, − 47.7, − 20.0), mothers having no education beta =  − 7.0 (− 14.8, 0.7), primary education beta =  − 14.6 (− 25.8, − 3.4), and secondary education beta =  − 11.2 (− 19.7, − 2.8) compared to those with educational status of higher secondary and above. A significant decline was also observed in those having household monthly income of < USD 53 beta =  − 30.5 (95% CI, − 42.3, − 18.7), USD 53–159 beta =  − 5.8 (95% CI, − 11.5, − 0.1), and USD 159–320 beta =  − 10.5 (95% CI, − 18.8, − 2.1) compared to those with an income of > 320 USD. Conversely, the processing speed scores were significantly increased among those who underwent surgical tumor resection, i.e., total resection beta =  − 30.5 (95% CI, − 42.3, − 18.7), subtotal resection beta =  − 5.8 (95% CI, − 11.5, − 0.1), and maximum safe resection beta =  − 10.5 (95% CI, − 18.8, − 2.1) compared to those who underwent no surgical resection (Table 4, Supplementary 2).

## Discussion

The studies conducted in high-income countries as reported in a narrative review by Stavinoha et al. are largely cross-sectional or, where longitudinal, without a clearly defined baseline [14]. However, from the Asian context, a systematic review of 59 studies by Poon et al. [17] reported long-term outcomes of lymphoblastic leukemia and CNS tumors survivors, and only one study assessed the neurocognition outcomes reporting impairment in 10% of CNS tumor survivors [18]. From Pakistan, only one study assessed the neurological, endocrine, hypothalamic complications and survival outcomes in children with craniopharyngioma. However, this study did not assess the neurocognitive outcomes [19]. Therefore, this prospective cohort study aimed to identify the factors associated with neurocognitive impairment among children and young people (CYP) with primary brain tumor after treatment in Pakistan.

The study found an association of tumor type with neurocognition outcomes in CYP with PBTs. There was a significant decline in processing speed scores in patients with glioblastoma (grade 4) and ependymoma (grades 2 and 3) compared to pilocytic astrocytoma (grade 1). These findings align with those reported by Klein et al. who found a higher prevalence of cognitive impairment in malignant brain tumors (grades 3 and 4) compared to low-grade tumors, independent of tumor volume, patient’s age, and tumor location [30]. The infiltrative and invasive nature of malignant tumors may impede neural plasticity by releasing tumor humoral substances that impairs cortical function, potentially causing decline in processing speed of the brain [31].

A statistically significant decline in processing speed was also observed in those with craniopharyngiomas compared to those with pilocytic astrocytomas. This is consistent with the findings by Merchant et al. who observed attention-related challenges in this group [32]. Craniopharyngiomas are histologically benign but, given their anatomical position in the sella turcica, frequently involve compression of adjacent critical structures such as the hypothalamus, pituitary glands, cranial nerves, and circle of Willis [33]. These tumors usually cause prominent emotional, cognitive, and behavioral disturbances derived from hypothalamic dysfunction [34]. Its proximity to the pituitary may also lead to endocrine irregularities [35]. Endocrinopathy plays a significant role in cognitive outcomes in craniopharyngioma too [36]. Although the study did not assess the endocrine profile of the patients, but a study from Pakistan reported that 37 (75%) patients with craniopharyngioma were found to have panhypopituitarism and required long-term hormonal replacement therapy 1 year after surgery [19].

This study also found a significant improvement in processing speed scores of those who underwent surgical resection as compared to those who did not undergo surgical resection. The current literature suggests that surgical intervention can mitigate various issues arising from tumor including relief of mass-related pressure and a reduction of swelling that leads to faster restoration of normal brain function and improvement in cerebrospinal fluid flow [37, 38]. This is consistent with Hendrix et al., who reported a low risk of cognitive deterioration following surgical resection. The neurocognitive function appeared to recover within the initial 2 months after the surgery in their sample [38]. Teixidor et al. also reported long-term improvement of verbal memory, after a transient immediate postoperative worsening, following surgical resection [39].

In this study, CYP with post-treatment seizures had a statistically significant decline in verbal and non-verbal neurocognitive scores. Seizures may have an impact on brain maturation and cognitive function in the CYP, the most vulnerable period of brain development [40, 41]. The study findings were consistent with a study by Phillips et al. which suggested that resolution of seizures among patients with CNS tumors lead to an improvement in neurocognition outcomes [42].

There was statistically significant decline in verbal and non-verbal neurocognitive domains among CYP with lower socioeconomic status, particularly in cases where the patients’ mothers had low or no formal education. These findings are consistent with Ellenberg et al., Ward et al., and Carlson-Green et al. all of which emphasized that environmental factors, including socioeconomic status (SES), loss of schooling and socialization, and alterations in family psychosocial functioning, can have an impact neurocognitive outcomes [43, 44]. A study by Nathan et al. also showed that parental education is a predictor of neurocognition outcomes [45]. It is possible that many children from higher (SES) families benefit from greater parental support, more cognitively stimulating home environments, and increased ability of parents to advocate for the unique needs of their children. Furthermore, it is reasonable to assume that more affluent educational institutions possess better resources to offer specialized assistance to children struggling with treatment-induced neurocognitive impairments. This suggests a role for educational infrastructure in addressing such challenges and underscores the need for equitable access to these resources across all segments of society [46].

### Study strengths

There were several strengths of the study: Firstly, the longitudinal study design with baseline information of the patients helped examining the neurocognition changes over time within the same group of patients. Secondly, study employed validated neurocognition assessment tools, ensuring the reliability and accuracy of the data collected. Thirdly, the psychologist devised a treatment plan for those CYP who were identified having a significant decline in 12 months post-treatment neurocognition scores. Lastly, given the similarity between those who were lost to follow-up at the 12 months post-treatment with those who were reassessed at that point, therefore attrition bias is unlikely to explain the findings (Supplementary 3).

### Study limitations

The study had a number of limitations. Firstly, the relatively small sample size restricted its statistical power. Secondly, the CYP population displayed heterogeneity with respect to tumor histopathology. Thirdly, the assessment was performed at only two time points, pre-treatment and at 12 months post-treatment: multiple assessments could have yielded more informative results. Furthermore, we did not evaluate the endocrine profile of the patients, a crucial aspect of adverse late effects and disturbances of which could mediate outcome. Fourthly, there was a higher proportion of females among those who were lost to follow-up compared to males. Lastly, we cannot exclude a degree of selection bias as those with operable conditions may naturally have more favorable long-term outcomes.

## Conclusions

In this novel study, the post-treatment mean change in verbal and non-verbal neurocognition scores was associated with sociodemographic, tumor, and treatment factors. These findings may have potential implications for targeted early psychological screening of higher risk CYP with PBTs. Identification of these predictors may serve as a foundation for developing more cost-effective treatment thereby alleviating the burden of neurocognitive morbidity. However, to establish generalizability, future research should prioritize larger-scale, multicountry studies.

## Study implications

A crucial aspect highlighted by the study is identifying factors associated with neurocognitive outcomes in the post-operative phase that would allow timely interventions in CYP with PBTs. This early response has the potential to prevent or mitigate cognitive decline, ultimately leading to better long-term outcomes for CYP affected by PBTs. By understanding the specific neurocognitive outcomes, clinicians can tailor interventions to address cognitive deficits, thus facilitating the development of personalized treatment plans that would enhance overall well-being. However, to achieve these goals, a sustained collaboration is imperative among various stakeholders, including psychologists, neuropsychologists, caregivers, rehabilitation facilities, and schools. This collaborative effort spans from the initial diagnosis to long-term follow-ups, ensuring a comprehensive and cohesive approach to the well-being of these children.

### Supplementary Information

Below is the link to the electronic supplementary material.

Supplementary file1 (DOCX 62 KB)

Supplementary file2 (DOCX 24 KB)

Supplementary file3 (DOCX 37 KB)

## Data Availability

The data that support the findings of this study are available from the corresponding author upon reasonable request.

## Abbreviations

CYP: Children and young people
PBT: Primary brain tumor
WHO: World Health Organization
QoL: Quality of life
CNS: Central nervous system
AKUH: Aga Khan University Hospital
JCIA: Joint Commission International Accreditation
JPMC: Jinnah Postgraduate Medical Centre
ADHD: Attention deficit hyperactivity disorder
PC: Proportionate change in means
CV: Coefficient of variation
SITS-R3: Slosson Intelligence tool, revised 3rd edition
M: Mean
SD: Standard deviation
CVI: Content validity index
CVR: Content validity ratio
RPM: Raven’s Progressive Matrices
PSI: Processing Speed Index
WISC: Wechsler Intelligence Scale
WAIS: Wechsler Adult Intelligence Scale
ERC: Ethics review committee
IQR: Interquartile range
CI: Confidence interval
VPS: Ventriculoperitoneal shunt
*r*: Correlation coefficient
