# Supplementary figures and images for: Predictors of neurocognition outcomes in children and young people with primary brain tumor presenting to tertiary care hospitals of Karachi, Pakistan: a prospective cohort study
Source: Childs Nerv Syst. 2024 Feb 16;40(6):1707–19. doi: 10.1007/s00381-024-06306-x (PMC11111568; doi:10.1007/s00381-024-06306-x)

**Supplementary 1**

**Pretreatment verbal intelligence grades**


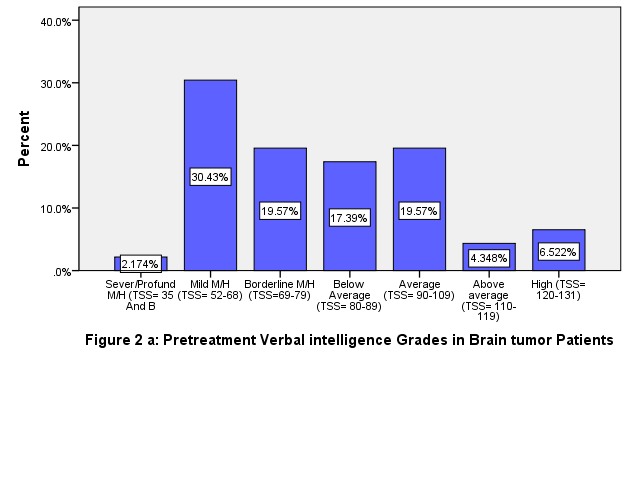


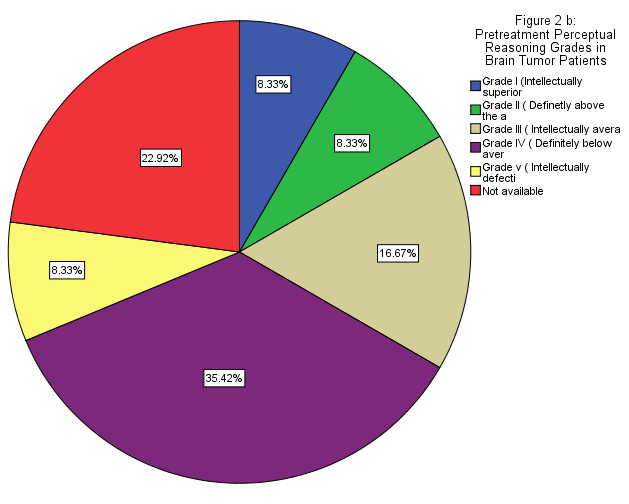

Supplement: Supplementary file 1 — Supplementary file1 (DOCX 62 KB) [file 381_2024_6306_MOESM1_ESM.docx]
