# Supplementary material for: Predictors of neurocognition outcomes in children and young people with primary brain tumor presenting to tertiary care hospitals of Karachi, Pakistan: a prospective cohort study
Source: Childs Nerv Syst. 2024 Feb 16;40(6):1707–19. doi: 10.1007/s00381-024-06306-x (PMC11111568; doi:10.1007/s00381-024-06306-x)
Supplement: Supplementary file 2 — Supplementary file2 (DOCX 24 KB) [file 381_2024_6306_MOESM2_ESM.docx]

**Supplementary 2**

**Factors associated with mean change in 12 months neurocognition scores in brain tumor patients.**

**3a. Mean change in verbal intelligence scores**

**3b. Mean change in perceptual reasoning scores**

**3c. Mean change in processing speed scores
**
