# Supplementary material for: Predictors of neurocognition outcomes in children and young people with primary brain tumor presenting to tertiary care hospitals of Karachi, Pakistan: a prospective cohort study
Source: Childs Nerv Syst. 2024 Feb 16;40(6):1707–19. doi: 10.1007/s00381-024-06306-x (PMC11111568; doi:10.1007/s00381-024-06306-x)
Supplement: Supplementary file 3 — Supplementary file3 (DOCX 37 KB) [file 381_2024_6306_MOESM3_ESM.docx]

**Supplementary 3**

**Risk of Bias Assessment**

| **Sociodemographic and birth related factors participants** | | | |
| --- | --- | --- | --- |
| **Factors** | **Followed for 12 months**  **(n=25)** | **Loss to follow-up**  **(n=13)** | **Died**  **(n=10)** |
| **Demographics Related n(%)** | | | |
| **Patient’s Age**  5-9  10-14  15-17  18-21  **Mean Age of the patients in years (SD)** | 08 (32)  06 (24)  06 (20)  05 (20)  13.2 (4.8) | 04 (31)  04 (31)  03 (23)  02 (15)  12.2 (4.8) | 03 (30)  04 (40)  02 (20)  01 (10)  12.1 (4.0) |
| **Gender***  Male  Female | 17 (68)  08 (32) | 05 (39)  08 (61) | 07 (70)  03 (30) |
| **Province of Residence**  Sindh  Punjab  Kyber Pakhtunkhwa  Gilgit Baltistan | 20 (80)  03 (12)  02 (08)  00 (0) | 08 (61)  03 (23)  01 (08)  01 (08) | 07 (70)  02 (20)  01 (10)  00 (0) |
| **Mother Tongue**  Sindhi  Urdu  Pushto  Punjabi  Others (Saraiki and Shina) | 03 (12)  15 (60)  02 (08)  02 (08)  03 (12) | 02 (15)  04 (31)  02 (15)  02 (15)  03 (24) | 02 (20)  03 (30)  01 (10)  04 (40)  00 (00) |
| **Educational status of the child**  Primary  Secondary  Higher secondary or above  **Median Years of child’s formal education (IQR)** | 10 (40)  09 (36)  06 (24)  7 (1.5-10.5) | 08 (62)  03 (23)  02 (15)  3 (0.5-8.5) | 06 (60)  03 (30)  01 (10)  4.5 (1.8-9) |
| **Household Family Members**  ≤ 6  >6  **Median Number of Household Family Member (IQR)** | 13 (52)  12 (48)  06 (5-7.5) | 04 (31)  09 (69)  08 (5.5-11) | 04 (40)  06 (60)  07 (5-7) |
| **Number of Siblings**  ≤ 3  >3  **Median Number of Siblings (IQR)** | 18 (72)  07 (28)  03 (2-4) | 07 (54)  06 (46)  03 (2-4) | 06 (60)  04 (40)  03 (2-5) |

| **Birth Related Factors of the patient n(%)** | | | |
| --- | --- | --- | --- |
| **Gestational age ****  **Preterm**  **Term**  **Mean Gestational age in weeks (SD)** | 06 (24)  19 (76)  36.7 (2.7) | 01 (08)  12 (92)  37.7 (0.7) | 00 (0)  10 (100)  38.0 (38-38) |
| **Birth Order**  First  Middle  Last  **Median Birth order of the child (IQR)** | 08 (32)  13 (52)  04 (16)  02 (1-3) | 04 (31)  05 (39)  04 (31)  02 (1-4) | 03 (30)  07 (70)  00 (0)  2.5 (1-3.3) |
| **Parental Sociodemographic n(%)** | | | |
| **Age of Mother (in year)**  25-34  ≥ 35  **Mean age in years (SD)** | 07 (28)  18 (72)  38.6 (6.4) | 02 (15)  11 (85)  38.9 (6.5) | 07 (70)  03 (30)  32.5 (30-39.8) |
| **Age of Father (in years)**  25-34  ≥ 35  **Mean age in years (SD)** | 04 (16)  21 (84)  42.7 (8.2) | 01 (08)  12 (92)  43.5 (9.3) | 00 (0)  10 (100)  41.5 (37.5-46.3) |
| **Marital status**  Married  Others (Widower, Widow, Divorce) | 21 (84)  04 (16) | 11 (85)  02 (15) | 10 (100)  00 (0) |
| **Educational status of Mother**  No formal education  Primary  Secondary  Higher Secondary and above  **Median Years of Mothers education (IQR)** | 05 (20)  03 (12)  05 (20)  12 (48)  10 (3-14) | 06 (46)  03 (23)  01 (08)  03 (23)  05 (0-11) | 01 (10)  03 (30)  02 (20)  04 (40) |
| **Education status of father**  No formal education  Primary  Secondary  Higher Secondary and above  **Median Years of Father education (IQR)** | 03 (12)  00 (0)  03 (12)  19 (76)  12 (11-16) | 05 (39)  02 (15)  02 (15)  04 (31)  05 (0-12) | 02 (20)  01 (10)  04 (40)  03 (30)  09 (5-12) |
| **Working status of the parents**  Only Father Working  Only Mother Working  Both Father and Mother Working  Both Father and Mother Not Working | 19 (76)  02 (08)  01 (04)  03 (12) | 11 (85)  00 (0)  01 (08)  01 (08) | 08 (80)  00 (0)  01 (10)  01 (10) |
| **Household Monthly income (in USD)**  ≤ 53  53-159  159-320  >320  **Median Monthly Income in USD (IQR)** | 04 (16)  09 (36)  04 (16)  08 (32)  141.3 (70.6-583) | 03 (23)  07 (54)  02 (15)  01 (08)  106 (61.8-220) | 01 (10)  07 (70)  01 (10)  01 (10)  106 (70.7-163.4) |
| *Current conversion rate of USD is 283*  **significant at p value <0.05 by using chi-square test*  ***Gestational age; Preterm defined as delivery before 37 weeks of gestation; term defined as delivery between 37+0 to 41+6 weeks* | | |  |

| **Tumor and Treatment related factors of participants with Brain Tumor** | | | |
| --- | --- | --- | --- |
| **Factors** | **Completed the follow-up (n=25)** | **Loss to follow-up (n=13)** | **Dead**  **(n=10)** |
| **Tumor related n(%)** | | | |
| **Patient’s age at diagnosis**  5-9  10-14  15-17  18-21  **Mean age of patient at tumor diagnosis in years (SD)** | 09 (36)  05 (20)  07 (28)  04 (16)  12.8 (4.8) | 05 (39)  03 (23)  03 (23)  02 (15)  12.1 (4.8) | 03 (30)  04 (40)  03 (30)  00 (0)  11.7 (3.2) |
| **Site of treatment**  Private Tertiary Care Hospital  Public Tertiary Care Hospital | 18 (72)  07 (28) | 06 (46)  07 (54) | 05 (50)  05 (50) |
| **Location of Brain Tumor**  Supratentorial  Cerebrum  Intraventricular  Infratentorial  Cerebellum  Brainstem  Fourth Ventricle  Suprasellar  Hypothalamus  Thalamus  Third ventricle  Infundibulum  Optic chiasma  Not known  Sellar  Pituitary  Multiple | 08 (32)  08  00  06 (24)  04  00  02  07 (28)  02  00  01  02  02  00  03 (12)  03  01 (04) | 03 (23)  01  02  06 (46)  04  01  01  02 (15)  00  01  00  00  00  01  01 (08)  01  01 (08) | 01 (10)  01  00  05 (50)  00  03  02  03(30)  02  01  00  00  00  00  01 (10)  01  00 (0) |
| **Histopathology of Tumor**  Glioblastoma  Medulloblastoma  Ependymoma  Diffuse Astrocytoma  Craniopharyngioma  Pilocytic Astrocytoma  Pituitary Adenoma  Others ^#^  Not known | 03 (12)  01 (04)  01 (04)  01 (04)  04 (16)  09 (36)  02 (08)  02 (08)  02 (08) | 01 (08)  01 (08)  02 (15)  00 (0)  00 (0)  03 (23)  02 (15)  01 (08)  03 (23) | 00 (0)  05 (50)  00 (0)  00 (0)  01 (10)  00 (0)  01 (10)  00 (0)  03(30) |
| **Grade of Tumor**  Grade 1  Grade 11  Grade 111  Grade 1V  Not known | 16 (64)  02 (08)  01 (04)  04 (16)  02 (08) | 06 (46)  01 (08)  01 (08)  02 (15)  03 (23) | 02 (20)  00 (0)  00 (0)  05 (50)  03 (30) |

| **Tumor Size (in mm^3^)**  ≤9135  9136 – 35088  35089 – 90000  >90000  Not available  **Median Tumor Size (IQR)** | 03 (12)  05 (20)  04 (16)  05 (20)  08 (32)  35742 (17413-96509) | 03 (23)  03 (23)  02 (15)  01 (08)  04 (31)  17255 (5790-48504) | 02 (20)  00 (0)  02 (20)  01 (10)  05 (50)  52875 (4680-154922) |
| --- | --- | --- | --- |
| **History of Seizure**  Yes  No | 07 (28)  18 (72) | 02 (15)  11 (85) | 01 (10)  09 (90) |
| **Hydrocephalus***  Present  Absent | 08 (32)  17 (68) | 09 (69)  04 (31) | 06 (60)  04 (40) |
| **Family History of Brain Tumor**  Yes  No | 03 (12)  22 (88) | 02 (15)  11 (85) | 00 (0)  10 (100) |
| **Family History of any other Cancer**  Yes  No | 05 (20)  20 (80) | 02 (15)  11 (85) | 04 (40)  06 (60) |
| **Treatment Related n(%)** | | | |
| **Use of steroid**  Yes  No | 20 (80)  05 (20) | 12 (92)  01 (08) | 09 (90)  01 (10) |
| **Post-treatment Seizures***  Yes  No  No information | 03 (12)  21 (84)  01 (04) | 01 (08)  00 (00)  12 (92) | 00 (0)  00 (0)  10 (100) |
| **Type of treatment**  Surgery only  Radiotherapy only  Combination  Surgery and Chemotherapy  Surgery and Radiotherapy  Surgery, Radiotherapy & chemotherapy  No intervention | 14 (56)  00 (0)  07 (28)  02  02  03  04 (16) | 09 (69)  00 (0)  01 (08)  00  00  01  03 (23) | 03 (30)  01 (10)  04 (40)  02  01  01  02 (20) |
| **Type of Surgery ****  Biopsy  Total resection  Subtotal resection  Maximum safe resection  No surgical intervention | 00 (0)  07 (28)  01 (04)  13 (52)  04 (16) | 01 (08)  03 (23)  01 (08)  05 (38)  03 (23) | 00 (0)  00 (0)  00 (0)  07 (70)  03 (30) |
| **Presence of VPS/ EVD**  Yes  Only EVD  Only VPS  EVD and VPS  No | 08 (32)  02  03  03  17 (68) | 09 (69)  02  05  02  04 (31) | 05 (50)  00  02  03  05 (50) |
| **Recurrence**  Yes  No  No information | 01 (04)  23 (92)  01 (04) | 00 (0)  02 (15)  11 (85) | - |
| #Histopathology for others include; anaplastic astrocytoma, optic chiasma and choroid plexus papilloma  **Signifciant at p value < 0.05 by using fisher exact test*  ***Type of Surgery: total resection (100% of tumor removal); maximum safe resection (> 90% of tumor removal); subtotal resection (< 90% of tumor removal)*  *EVD; External ventricular drain, VPS; ventriculoperitoneal shunt* | | | |

| **Pretreatment Neurocognition Scores of participants with Brain Tumor** | | | |
| --- | --- | --- | --- |
|  | Followed for 12 months (n=25) | Loss to follow-up  (n=13) | Dead  (n=10) |
| **Neurocognition domains** | **Mean Pretreatment scores (SD)** | | |
| **Verbal Intelligence Scores** | 85.8 (19.9) | 72.9 (17.3) | 79.6 (18.7) |
| **Perceptual Reasoning Scores** | 94.5 (14.7) | 90.7 (14.8) | 95.7 (18.9) |
| **Processing speed Scores** | 66.0 (13.97) | 64.5 (15.4) | 53.2 (10.8) |
